# Genome-Wide Characterization of Simple Sequence Repeat (SSR) Loci in Chinese Jujube and Jujube SSR Primer Transferability

**DOI:** 10.1371/journal.pone.0127812

**Published:** 2015-05-22

**Authors:** Jing Xiao, Jin Zhao, Mengjun Liu, Ping Liu, Li Dai, Zhihui Zhao

**Affiliations:** 1 Research Center for Chinese Jujube, Agricultural University of Hebei, Baoding, 071000, China; 2 College of Life Science, Agricultural University of Hebei, Baoding, 071000, China; National Institute of Plant Genome Research (NIPGR), INDIA

## Abstract

Chinese jujube (*Ziziphus jujuba*), an economically important species in the Rhamnaceae family, is a popular fruit tree in Asia. Here, we surveyed and characterized simple sequence repeats (SSRs) in the jujube genome. A total of 436,676 SSR loci were identified, with an average distance of 0.93 Kb between the loci. A large proportion of the SSRs included mononucleotide, dinucleotide and trinucleotide repeat motifs, which accounted for 64.87%, 24.40%, and 8.74% of all repeats, respectively. Among the mononucleotide repeats, A/T was the most common, whereas AT/TA was the most common dinucleotide repeat. A total of 30,565 primer pairs were successfully designed and screened using a series of criteria. Moreover, 725 of 1,000 randomly selected primer pairs were effective among 6 cultivars, and 511 of these primer pairs were polymorphic. Sequencing the amplicons of two SSRs across three jujube cultivars revealed variations in the repeats. The transferability of jujube SSR primers proved that 35/64 SSRs could be transferred across family boundary. Using jujube SSR primers, clustering analysis results from 15 species were highly consistent with the Angiosperm Phylogeny Group (APGIII) System. The genome-wide characterization of SSRs in Chinese jujube is very valuable for whole-genome characterization and marker-assisted selection in jujube breeding. In addition, the transferability of jujube SSR primers could provide a solid foundation for their further utilization.

## Introduction

Microsatellites, or simple sequence repeats (SSR), are iterations of between 1- and 6-bp nucleotide motifs. These sequences have been detected in the genomes of numerous organisms and are distributed throughout the entire genome in both coding and non-coding regions [1, 2]. Given their many desirable attributes, including wide genomic distribution, co-dominant inheritance, their multi-allelic nature, and a high level of polymorphisms, SSRs are highly favored molecular markers [3–5]. SSRs are also very useful in genetic analysis, molecular assisted breeding, genetic mapping, and varietal identification [6–8]. Moreover, SSRs are easily assayed by PCR [9]. As next-generation sequencing technology has developed, *de novo* genome sequencing has greatly accelerated SSR discovery and numerous additional SSR loci could be identified using genome-wide sequence analysis.

The Chinese jujube (*Ziziphus jujuba* Mill.), which belongs to the Rhamnaceae family, originated in China and has been cultivated for more than 7,000 years [10]. This tree has been introduced into approximately 50 countries throughout the world, including Japan, Korea, India, Tunisia, Italy, the United States, and Australia. Approximately 900 Chinese jujube cultivars are available [11]. Previously reported jujube SSR markers were proved to be very useful in genetic analysis of jujube and wild jujube [12–14], and more available SSR markers should be developed. In addition, no reports have characterized SSRs throughout the Chinese jujube genome.

We recently sequenced the genome of the Chinese jujube *de novo* [15]. The objectives of this study included the following: (a) to perform genome-wide characterization of SSRs in the jujube genome, (b) to develop and evaluate jujube SSR primers, and (c) to determine the transferability of jujube SSR primers to a wide range of angiosperm families. To our knowledge, this is the first report characterizing genome-wide SSRs in the Chinese jujube and the transferability of jujube SSR primers. This study will provide a foundation for the further utilization of jujube SSR primers.

## Results and Discussion

### Characterization of jujube SSRs

Using the MISA program to analyze 396.18 Mb (approximately 90.00%) of the estimated jujube genome [13], 70.83% of the 3,027 scaffold sequences were found to contain SSR loci. A total of 436,676 SSR loci were identified, with an average distance of 0.93 Kb between the loci. Over two-thirds (67.62%) of the scaffold sequences contained more than one SSR. Among the 480 types of motifs that were identified, mononucleotide and dinucleotide repeats were the most common in the intronic, UTR and non-genic regions, and trinucleotide repeats were the most prevalent type in the exonic region (S1 Table). Among the different types of repeats, mononucleotide repeats (283,301) were the most common, accounting for 64.87% of all repeats, followed by dinucleotides (24.40%), trinucleotides (8.74%), tetranucleotides (1.64%), pentanucleotides (0.21%), and hexanucleotides (0.14%).

Numerous SSRs were identified in the jujube genome, and their primitive characteristics were consistent with those of many other plant genomes, such as apple [16] and grape [17]. Species with a large number of short repeat-type SSR loci generally exhibit a higher genomic mutation rate [18–20]. The high proportion of short repeat-type SSR loci in the jujube genome indicates that this genome has a long evolutionary history or that it has a high mutation rate.

The mononucleotide repeats exhibited a strong bias toward A/T motifs (98.48%) compared with C/G repeats (Table 1). The AT/AT motif (74.33% in total dinucleotide repeats) was the most common type, whereas CG/CG was present at very low levels (0.02%). Among the other types of repeats, the most prevalent included AAT/ATT (64.17%), AAAT/ATTT (79.45%), AAAAT/ATTTT (51.23%), and AAAAAG/CTTTTT (52.96%). Jujube SSR repeat motifs exhibited a preference for A and T, which is consistent with the results from a previous study of a small region of the jujube genome (8.4 Mb) [21]. That study also indicated that hexanucleotide repeats were the most abundant, which is inconsistent with our results. This difference suggested that whole-genome sequencing is necessary for SSR characterization.

**Table 1:** SSR frequency in the jujube genome.

SSR Motif	Number of Repeat Units												Total
	5	6	7	8	9	10	11	12	13	14	15	>15	
A/T	-	-	-	-	-	69067	39804	30597	25125	21449	18879	74082	279003
C/G	-	-	-	-	-	676	510	446	414	337	305	1610	4298
AT/AT	-	-	10521	12065	14460	14498	11745	7287	3854	2016	1031	1715	79192
AG/CT	-	-	3253	2604	2250	2065	1651	1384	1144	1013	763	203	19583
AC/GT	-	-	1391	1201	1010	846	662	533	422	342	262	1068	7737
CG/CG	-	-	17	7	-	1	-	-	-	-	-	-	25
AAT/ATT	10202	6080	3603	1900	1084	597	357	213	158	87	59	153	24493
AAG/CTT	3208	1718	989	554	318	210	106	55	33	20	14	46	7271
ACT/AGT	1044	595	339	158	77	49	28	19	6	5	3	14	2337
AAC/GTT	857	609	295	121	78	55	31	9	8	6	4	9	2082
ACC/GGT	465	200	117	48	32	8	11	4	2	2	-	2	891
AGG/CCT	286	121	58	41	20	10	7	1	2	1	-	-	547
ACG/CGT	311	90	47	20	5	3	2	1	1	-	-	-	480
CCG/CGG	48	13	4	2	-	-	-	-	-	-	-	-	67
AAAT/ATTT	4539	903	166	48	8	5	1	-	2	-	-	4	5676
AAAG/CTTT	291	128	58	32	9	5	3	2	-	-	-	1	529
AATT/AATT	180	55	10	3	-	-	-	-	-	-	-	1	249
AACT/AGTT	122	49	30	12	6	7	7	4	-	1	1	10	241
AAAC/GTTT	140	51	14	6	2	-	-	-	-	-	-	1	214
AAAAT/ATTTT	406	61	5	4	1	1	-	-	-	-	1	-	479
AAAAG/CTTTT	120	19	6	2	-	-	1	-	-	-	-	-	148
AAAAC/GTTTT	60	13	4	1	1	-	-	-	-	-	-	-	79
AAAAAG/CTTTTT	222	63	15	6	-	-	2	-	-	-	-	5	313
AAAAAT/ATTTTT	70	11	1	1	-	-	-	-	-	-	-	-	83

### Comparison of genomic SSRs from jujube with other species

The jujube genome is smaller than the apple, pear, and grape genomes, but it contains more SSRs (S2 Table). The average distance between SSRs varied between the species, with the smallest distance (2.65 Kb) noted in jujube and the largest (7.52 Kb) in apple (S2 Table). The jujube genome exhibited the highest SSR density (387 SSRs/Mb) followed by mulberry (281 SSRs/Mb), peach (219 SSRs/Mb), and *Prunus mume* (211 SSRs/Mb). Overall, the jujube genome contains significantly more SSR loci compared with the other seven species.

The predominant SSR motifs differ among the different species (S3 Table). AT/AT was the primary dinucleotide motif in jujube, grape, and mulberry, whereas the AG/CT motif was the most common in peach, strawberry, and *Prunus mume*. In addition, a large proportion of both motifs were noted in the apple and pear genomes. The predominant trinucleotide repeats included AAT/ATT in jujube, grape, and mulberry; AAG/CTT in strawberry; AAC/GTT and AAG/CTT in apple; and AAT/ATT and AAG/CTT in pear, peach, and *Prunus mume*. Among the tetranucleotide repeats, AAAT/ATTT was the most common motif in all tested species. Although the most common SSR motifs varied among species, all of the dominant repeat motifs were A/T rich, which is consistent with previous results regarding eukaryotic SSR loci [18]. This phenomenon might result from the conversion of cytosine to thymine [22]. In monocotyledons, CCG/CGG is the primary trinucleotide repeat motif, but this motif is very rare in dicotyledons [23]. It is possible that trinucleotide repeats have a high GC content in monocotyledons [24]; alternatively, this phenomenon is potentially caused by a preference for certain bases [25].

### Screening of jujube SSR primers

In total, 283,301 mononucleotide repeats were excluded, and the remaining 153,375 SSRs were used to design primers. Primer pairs were successfully designed for 78,928 SSRs. Then those primer pairs with product size between 120 and 280 bp in length were selected, we reduced this number to 46,314 primer pairs.

In SSR loci with > 60-bp repeats, the amplification efficiency of the primers and the proportion of polymorphisms were significantly reduced compared with those for SSR loci ≤ 60 bp (Table 2). Theoretically, longer SSR loci are more polymorphic, but our results indicate that the SSR locus length is not positively correlated with the proportion of polymorphisms. This result was supported by previous studies, which also reported a balance between an increasing SSR locus length and an increasing proportion of polymorphisms without the need of a positive correlation [12, 26–27]. Then, we focused on SSR loci containing ≤ 60-bp repeats, and 30,565 primer pairs were further screened. Among them, dinucleotide and trinucleotide repeats were the most common, accounting for 81.48% and 13.39% of the amplicons, respectively. Furthermore, 1,000 primer pairs were randomly selected from these 30,565 pairs (S4 Table) and were evaluated using 6 jujube cultivars and wild types. Of the initial 1,000 primer pairs, 725 were shown to be effective, and 511 were polymorphic (S5 Table). The most common motifs were dinucleotide (243 primers) and trinucleotide repeats (232 primers).

**Table 2:** The polymorphic proportion and amplification efficiency of primers for different types of motifs.

Motifs types	Length	Number of tested primers	Number of effective primers	Proportion of effective primers (%)	Number of polymorphic primers	Proportion of polymorphic primers (%)
Dinucleotide	> 60bp	15	10	66.67	1	10.00
	≤ 60bp	15	13	86.67	7	53.85
Trinucleotide	> 60bp	15	5	33.33	1	20.00
	≤ 60bp	15	13	86.67	10	76.92
Tetranucleotide		6	4	66.67	2	50.00
Pentanucleotide		6	4	66.67	1	25.00
Hexanucleotide		8	5	62.50	1	20.00
Total		80	54		23	

Note: Effective primers mean the primers which amplified successfully in the size expected for jujube cultivars. Proportion of effective primers (%) = Number of effective primers/Number of tested primers × 100%

**Fig 1:**
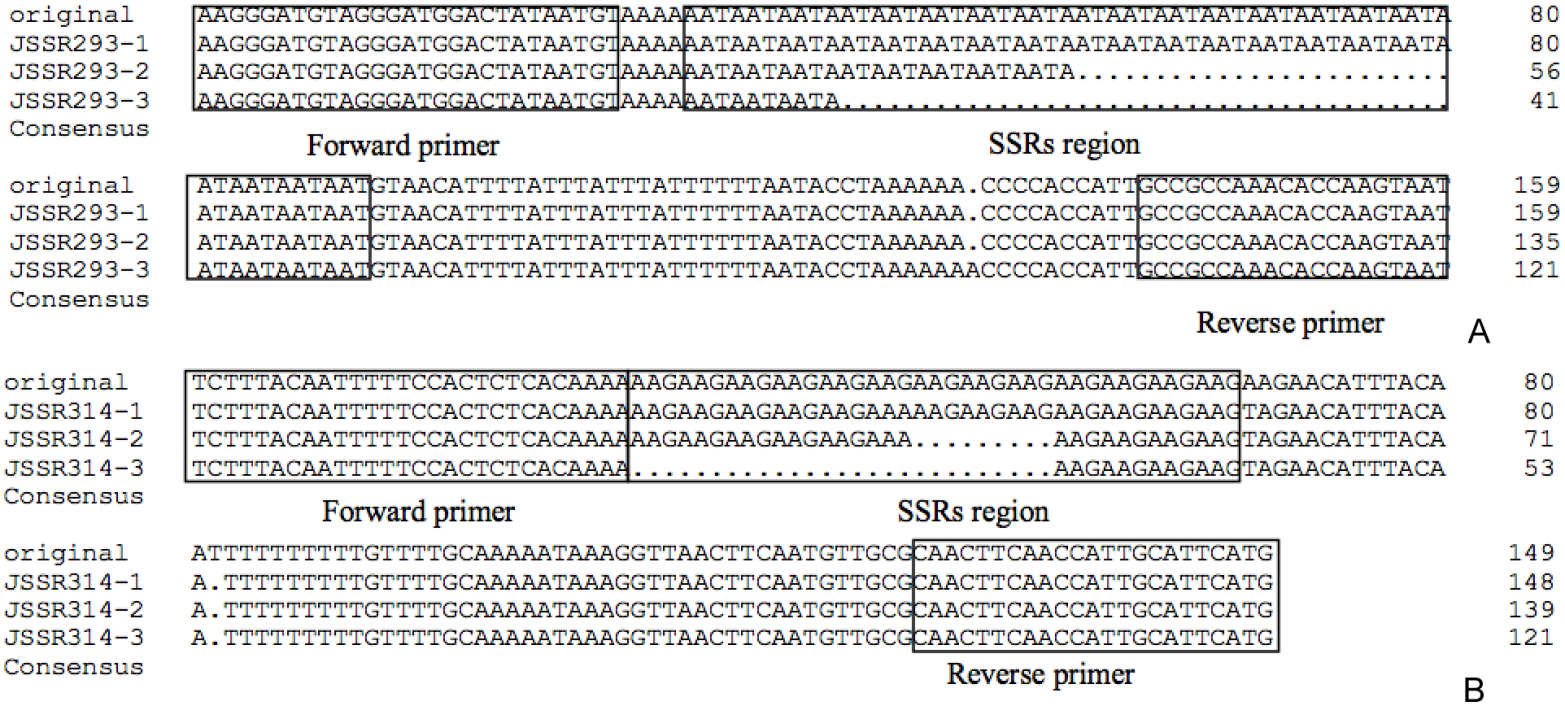
The verification and allelic diversity of jujube SSR loci. These sequence alignments of three jujube cultivars amplified by JSSR293 (A) and JSSR314 (B) were performed by DNAMAN. Allelic variation was detected at AAT repeats (JSSR293) and AAG repeats (JSSR314).

Primers for different types of motifs should exhibit different effects on the proportion of polymorphisms and the amplification efficiency. Because only a limited number of tetranucleotide, pentanucleotide, and hexanucleotide repeats were identified, we focused on the amplification effects of primers for dinucleotide and trinucleotide repeats. Increased proportions of effective primers and polymorphic amplicons were observed for dinucleotide repeats and trinucleotide repeats, respectively (Table 2). The lower proportion of polymorphisms in dinucleotide repeats indicates that this type of SSR locus is more highly conserved in the genome to ensure species stability. The higher polymorphic rate in trinucleotide repeats was similar to previous study [13], and that might be attributed to three bases coding for an amino acid, and frameshift mutations could effectively prevent other SSR loci from changing. From the perspective of SSR evolutionary analysis, these changes in the SSR loci could be explained with a stepwise mutation model (SMM); the length of an SSR locus was changed by adding or removing a percentage of the motif, not by adding or removing a single base [28]. This viewpoint was confirmed by sequencing (Fig 1).

The sequencing and alignment of the jujube SSR alleles verified the presence of SSR loci and revealed a high degree of conservation of the regions flanking the SSRs (Fig 1). The sequencing results also indicated that the loci had diverse alleles, which may contribute to the diversity of the jujube germplasm. The allelic diversity was mainly due to variations in microsatellite repeat lengths combined with point mutations within the flanking regions.

### Polymorphic verification of jujube SSR markers

**Fig 2:**
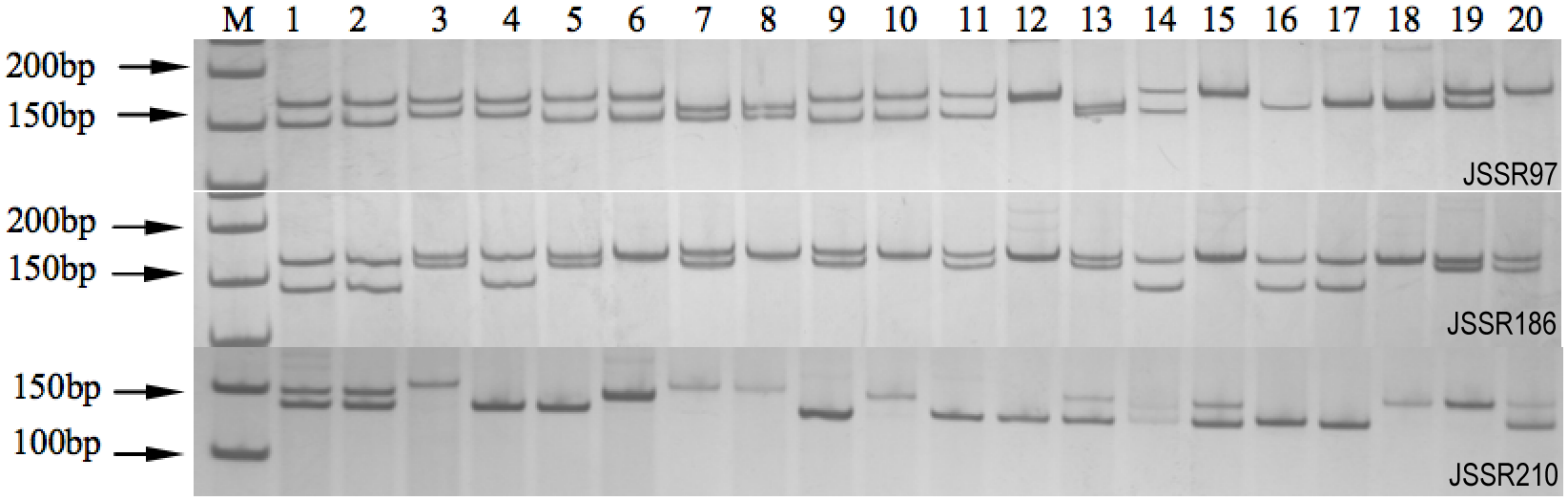
Amplification products from 20 jujube cultivars using the JSSR97, JSSR186, and JSSR210 primers. M: 50-bp marker; numbers 1 to 20: 20 jujube cultivars listed in Table 4.

Among the 511 polymorphic SSR primers, 16 pairs were randomly selected and used to analyze the genetic relationship among 20 jujube cultivars (Fig 2, Table 3). The primers amplified 68 polymorphic bands, and each primer pair amplified an average of 4.25 polymorphic fragments. The polymorphism information content (PIC) values of the loci were between 0.51 and 0.72, with an average of 0.61. Loci with PIC > 0.5 were considered highly polymorphic [29]. Therefore, 16 primer pairs were highly efficient.

**Table 3:** Information on the 16 jujube SSRs and their polymorphism among the 20 jujube cultivars.

Primer name	Repeat motifs	Primer sequences (5’-3’)	T_m_ (°C)	Length of production (bp)	The number of polymorphic loci	PIC
JSSR88	(TACA)_37_	F: tcaataattccagccgaatcctta; R:tgggagtctagcttcattcaaaca	53	180	5	0.59
JSSR93	(TTG)_13_	F: ggaaggactttgtcagcatggtag; R:aacagcatatttggatccatttcg	53	155	5	0.72
JSSR95	(TTG)_11_	F: cggtgagagacattttgttggatt; R:ttccttactttcccaccttgttca	55	152	5	0.71
JSSR97	(TTG)_9_	F: gtccaaaggcccaacttctttagt; R:aggggactactcctctgctgagat	57	155	4	0.61
JSSR129	(TGG)_9_	F: tgctaatgaaaggaactctgggtc; R:tgatgggtatgaagaagcatcaga	55	158	4	0.60
JSSR131	(TGC)_11_	F: gtcacgctaaaaaccattacctgc; R:cacacttgggttttgatcccatac	57	151	4	0.66
JSSR177	(GAA)_11_	F: atagctgcgaagtgtttctaagcg; R:atgccagcgatggaaaatttaag	53	238	3	0.57
JSSR186	(CTT)_12_	F: aggcagtgagtttctgtgaccttt; R:ttcttgatggccttcatatcaaca	57	160	4	0.62
JSSR194	(CTA)_16_	F: ccaccaactttcgctacaacttct; R:caactaggtaggaaaacaaaaacagtgg	59	158	6	0.63
JSSR210	(CAC)_13_	F: tcgtccatgtataatttcaccacc; R:tgtccaaacctaaaagagataaaggc	57	154	3	0.55
JSSR211	(CAC)_12_	F: atcaagtaccgcaagagaagtgct; R:ttctcaactctctccttggcctta	60	158	4	0.67
JSSR222	(ATT)_17_	F: gcagctggatgagaaccataa; R:acaatacaatacaaagccacattagttc	57	146	5	0.51
JSSR239	(ATG)_12_	F: gcaagtaccatacacaggatacgtc; R:gcataaagtttgtggaaaacgtaattt	57	158	4	0.51
JSSR244	(ATC)_15_	F: cactgcaaatgctttgtcatcttt; R:aaagcatcacccatcctctacatc	57	120	5	0.66
JSSR262	(ATA)_9_	F: cgtggaccaagtctataccaaaatg; R:tggtttttcttctcctaatccatgtg	57	240	3	0.56
JSSR438	(AG)_28_	F: tcggattgtataaatgggatttcg; R:tgtcacccaaaataccttctcttttt	57	230	4	0.64
Total					68	
Mean					4.25	0.61

**Fig 3:**
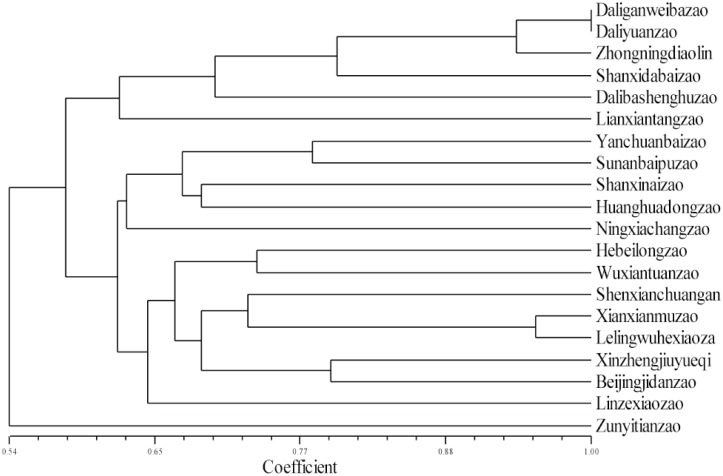
Dendrogram of 20 jujube cultivars based on 16 SSRs. 16 SSRs are listed in Table 3, and the 20 jujube cultivars are listed in Table 4.

The genetic relationship among the 20 jujube cultivars was constructed in a dendrogram using Numerical Taxonomy System of Multivariate Programs (NTSYS) cluster analysis (Fig 3). Both ‘Daliganweibazao’ and ‘Daliyuanzao’ are from Dali County, and these cultivars exhibit similar botanical characteristics and fruit shapes. The two cultivars were not separated in the dendrogram, indicating that they are either the same cultivar or they have a very close relationship. The genetic similarity coefficient between ‘Zunyitianzao’ (from Southern China) and the other 19 cultivars (from Northern China) was considerably low, which is consistent with their distant geographical locations. The related study among sour jujube populations also provided valuable information about genetic diversity and geographical distances [14].

### The transferability of jujube SSR primers

**Fig 4:**
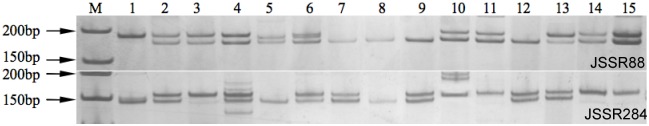
Amplification products from 15 species using the JSSR88 and JSSR284 primers. M: 50-bp marker; numbers 1 to 15: Arabidopsis, eggplant, tomato, wheat, corn, Chinese cabbage, cotton, grape, apple, peach, strawberry, pear, wintersweet, jujube, and wild jujube.

To verify the transferability of the primers to 15 species from 8 angiosperm families, 64 pairs of jujube SSR primers (S6 Table) were randomly selected from the 1,000 pairs mentioned above, and 35 pairs of SSR primers yielded polymorphisms (S7 Table, Fig 4). The primers amplified 107 polymorphic bands. Each primer pair amplified an average of 3.06 polymorphic fragments, and the PIC values ranged from 0.20 to 0.70, with an average of 0.48. Overall, 100% of the 64 primers successfully produced amplification products in wild jujube. Moreover, 65.63% (42/64), 39.06% (25/64), 35.94% (23/64), 37.50% (24/64), 25.00% (16/64), 20.31% (13/64), and 29.69% (19/64) of the 64 primers successfully produced amplification products in the Rosaceae family and the Vitales, Brassicales, Solanales, Malvales, Laurales, and Poales orders, respectively.

**Fig 5:**
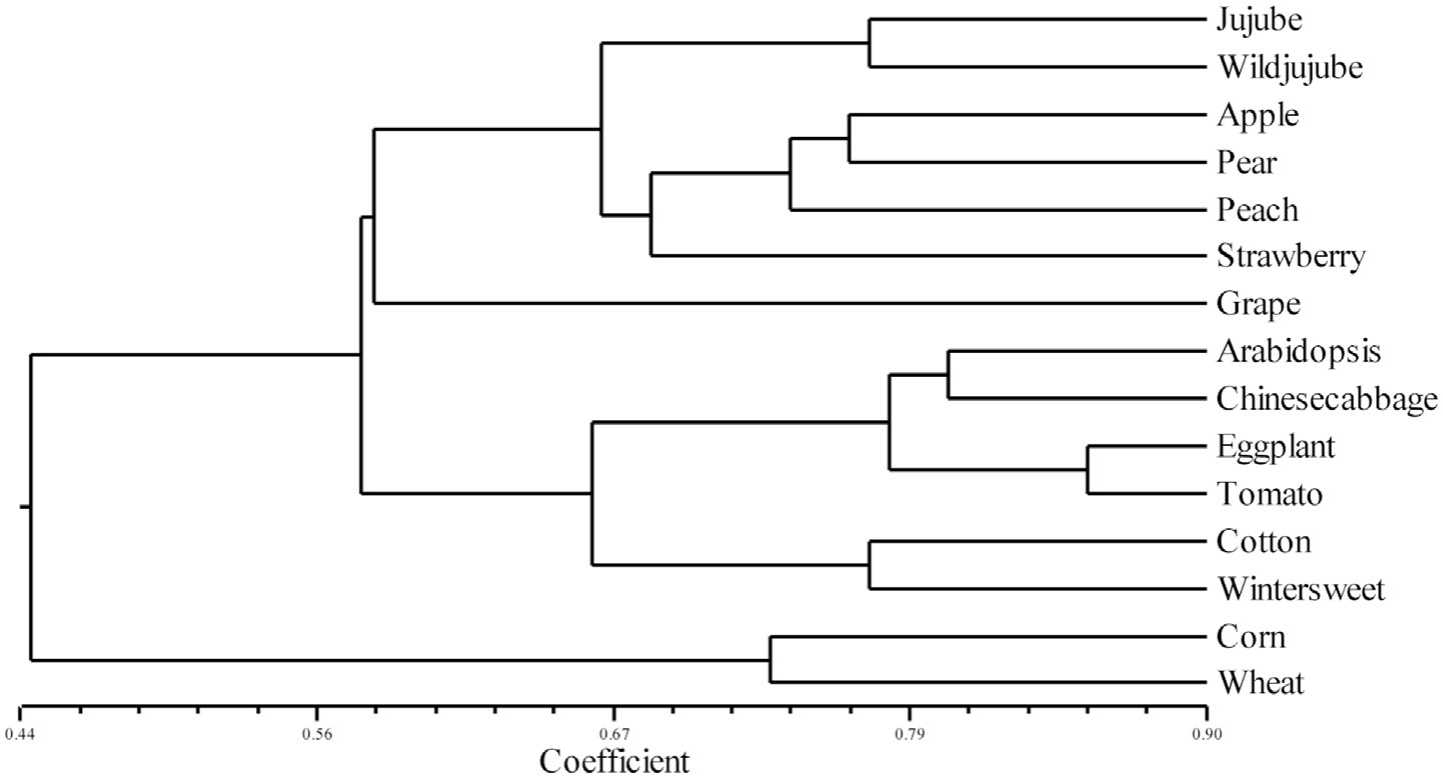
Dendrogram of 15 species based on SSR analysis. Numbers 1 to 15: Arabidopsis, eggplant, tomato, wheat, corn, Chinese cabbage, cotton, grape, apple, peach, strawberry, pear, wintersweet, jujube, and wild jujube.

The 15 tested species grouped into two main clusters, one with the 13 dicotyledonous species and the other with the two monocotyledonous species (Fig 5). The clustering results indicated that wild jujube was closely related to the 4 species in the Rosaceae family. Moreover, the similarity coefficient between jujube and peach (0.69) was higher than that between jujube and the other species of Rosaceae. This result indicated that the jujube has a closer relationship with the peach, which is supported by genomic data analysis [15]. The results were also highly consistent with the widely accepted Angiosperm Phylogeny System (http://www.mobot.org/MOBOT/Research/APweb/welcome.html) and provided evidence supporting the transferability of the jujube SSR primers.

SSR primers from the apple genome are transferable to pear; both species belong to the Rosaceae family [16, 30]. Fan et al. verified the transferability of SSR primers from the pear genome to other species in the Rosaceae family [31]. The transferability of SSR primers to other species, such as loquat [32], grape [33], strawberry [34], citrus [35], sweet cherry [36], and lychee [37], has also been reported. However, the transferability of plant SSR primers is typically studied at the family level. Our study was among the first to demonstrate the transferability of SSR primers to different families, which provides evidence for the wider application of plant SSR primers.

## Conclusion

In this study, we conducted genome-wide characterization of SSRs in jujube and used SSR markers to determine the transferability of jujube SSR primers to a wide range of angiosperm families. By analyzing the distribution of SSRs in the jujube genome and comparing the SSR pattern among jujube and other related species, we concluded that the jujube genome is significantly enriched for SSR loci compared with seven other species. Jujube SSR primers are valuable for marker-assisted selection in breeding, and their wide transferability would also provide a foundation for their further utilization.

## Materials and Methods

### Plant materials

Four cultivars of Chinese jujube (*Z*. *jujuba* Mill. ‘Dongzao’, ‘Wuhefeng’, ‘Dalilongzao’, and ‘Maoboyan’) and two strains of wild jujube (*Z*. *acidojujuba* Cheng et Liu ‘Xingtai 0605’ and ‘Xingtai 16’) were used in the primary evaluation of the new jujube SSR primers. An additional 20 cultivars of Chinese jujube of varied origins (Table 4) were used to verify the efficiencies of the primarily screened SSR primers. A total of 15 angiosperm species from 8 families and 7 orders (Table 5) were used to explore the transferability of the jujube SSR primers. All leaf samples were collected from the jujube germplasm repository of the Agriculture University of Hebei.

**Table 4:** The 20 jujube cultivars used in this experiment.

No.	Cultivars	Origin (Province)	No.	Cultivars	Origin (Province)
1	Daliganweibazao	Shanxi	11	Lelingwuhexiaozao	Shandong
2	Daliyuanzao	Shanxi	12	Sunanbaipuzao	Jiangsu
3	Yanchuanbaizao	Shanxi	13	Wuxianshuituanzao	Jiangsu
4	Shanxidabaizao	Shanxi	14	Zhongningdiaolingzao	Ningxia
5	Shanxinaizao	Shanxi	15	Lianxiantangzao	Guangdong
6	Dalibashenghuzao	Shanxi	16	Linzexiaozao	Gansu
7	Hebeilongzao	Hebei	17	Ningxiachangzao	Ningxia
8	Shenxianchuanganhongzao	Hebei	18	Xinzhengjiuyueqing	Henan
9	Xianxianmuzao	Hebei	19	Beijingjidanzao	Beijing
10	Huanghuadongzao	Hebei	20	Zunyitianzao	Guizhou

**Table 5:** The 15 species used to study the transferability of jujube SSRs.

Class	Order	Family	Species
Dicotyledons	Brassicales	Brassicaceae	Arabidopsis
			Chinese cabbage
	Solanales	Solanaceae	Eggplant
			Tomato
	Malvales	Malvaceae	Cotton
	Laurales	Calycanthaceae	Wintersweet
	Rosales	Rosaceae	Apple
			Pear
			Peach
			Strawberry
		Rhamnaceae	Jujube
			Wild jujube
	Vitales	Vitaceae	Grape
Monocotyledons	Poales	Poaceae	Wheat
			Corn

### DNA extraction and analysis

Genomic DNA was extracted from young leaves of different jujube cultivars using an improved cetyltrimethyl ammonium bromide (CTAB) method [38]. After extraction, 5–10 μl of DNA solution was loaded on a 1.0% agarose gel to assess the sample quality. Then, the DNA quality and concentration were further assessed using a NanoDrop2000.

### SSR identification and primer design

Identification and localization of the SSR primers in the Chinese jujube genome were performed using MISA (MIcroSAtellite) software with Perl. The following search criteria were implemented: ≥ 10 repeat units for mononucleotides, ≥ 7 repeat units for dinucleotides, and ≥ 5 repeat units for tri-, tetra-, penta-, and hexanucleotides. Interrupted compound SSRs were also selected when the bases interrupting the two SSRs were ≤ 10 repeat units. Primer pairs were designed using PRIMER 3 and were based on the flanking sequences of the identified SSRs; all primers were synthesized by Sangon Biotech, Shanghai, China.

### Comparison of genomic SSRs between jujube and other plants

Genomic data from apple [39], pear [40], peach [41], strawberry [42], *Prunus mume* [43], mulberry [44], and grape [45] were downloaded from the NCBI database. The genomic SSRs of other species were searched using the same criteria as employed for Chinese jujube and were then compared with the SSRs from the jujube genome. Mononucleotides were generally not very informative [46] and thus were not included in this analysis.

### Polymerase chain reaction and fragment analysis

Polymerase chain reaction (PCR) was performed in a total volume of 12.5 μl containing 0.5 μl of 50 ng/μl genomic DNA, 6.3 μl of 2×Taq Master Mix (CWBIO), and 0.5 μl of 10 μmol/L each of forward and reverse primers. The reactions were performed using the following conditions: 94°C for 3 min; 30 cycles of 94°C for 30 s, 50–60°C for 30 s, and 72°C for 30 s; and a final step at 72°C for 10 min. Then, 3 μl of the PCR product and a 50-bp molecular size marker were loaded onto an 8% denaturing polyacrylamide (PAGE) gel in 1×TBE buffer, run at 200 V, and visualized using silver staining. SSR analysis was performed at least twice to confirm primer amplification.

### Sequencing of SSR PCR products for SSR locus verification

To verify both the presence and allelic variation of the SSR loci, PCR amplification products from two primer pairs (JSSR293 and JSSR314) were resolved in 2.0% agarose gel and purified by SanPrep Column DNA Gel Extraction Kit. The product ligated into the pMD19-T vector and sequenced by Sangon Biotech.

### Data analysis

Only the fragments that could be clearly scored were used in the data analysis. The genotypic data were analyzed using the unweighted pair-group method with arithmetic averaging (UPGMA) clustering using NTSYS [47]. The numbers of alleles per locus and PIC values were calculated [48].

## Supporting Information

S1 Table The number and proportion of SSRs in the jujube genome. This table lists the numbers and proportions of the 6 types of SSR repeats in the jujube genome.
(XLS)

S2 Table The number of SSR loci in the genomes of 8 species. This table lists the repeat types and genome sizes of jujube, apple, pear, grape, peach, strawberry, *Prunus mume*, and mulberry.
(XLS)

S3 Table Predominant types of SSR motifs in the genomes of 8 species. This table lists predominant motif types in jujube, apple, pear, grape, peach, strawberry, *Prunus mume*, and mulberry.
(XLS)

S4 Table The 1,000 randomly selected SSR primer pairs. This table lists the names, repeat motifs, and sequences of 1,000 jujube SSR primers.
(XLS)

S5 Table The polymorphic proportion and amplification efficiency of 1,000 jujube SSR primers. This table lists the polymorphic proportion and amplification efficiency of 488 dinucleotide primers, 419 trinucleotide primers, 54 tetranucleotide primers, 20 pentanucleotide primers, and 19 hexanucleotide primers.
(XLS)

S6 Table Information on the 64 primer pairs. This table lists the primer names, repeat motifs, primer sequences, and amplicon lengths.
(XLS)

S7 Table The polymorphisms of 35 pairs of SSR primers. This table lists the polymorphic locus numbers and PIC values for 35 jujube SSR primers.
(XLS)
